# Supportive therapies in the prevention of chemotherapy-induced febrile neutropenia and appropriate use of granulocyte colony-stimulating factors: a Delphi consensus statement

**DOI:** 10.1007/s00520-022-07430-7

**Published:** 2022-11-05

**Authors:** Vincenzo Adamo, Lorenzo Antonuzzo, Marco Danova, Michelino De Laurentiis, Paolo Marchetti, Carmine Pinto, Giovanni Rosti

**Affiliations:** 1 Department of Human Pathology, Scientific Direction of Oncology, University of Messina, A.O. Papardo, Messina, Italy; 2 Clinical Oncology Unit, Careggi University Hospital, Florence, Italy; 3 Department of Experimental and Clinical Medicine, University of Florence, Florence, Italy; 4 Department of Internal Medicine and Medical Oncology, ASST of Pavia, Pavia, Italy; 5 LIUC University, Castellanza, Varese, Italy; 6 Department of Breast and Thoracic Oncology, Istituto Nazionale Tumori IRCCS “Fondazione G. Pascale”, Napoli, Italy; 7 Istituto Dermopatico Dell’Immacolata, IDI-IRCCS, Rome, Italy; 8 Medical Oncology Unit, Comprehensive Cancer Centre, AULS-IRRCS Reggio Emilia, Reggio Emilia, Italy; 9 Medical Oncology Fondazione IRCCS Policlinico San Matteo, Viale Camillo Golgi, 19, 27100 Pavia, PV Italy

**Keywords:** Chemotherapy, Consensus, Febrile neutropenia, Granulocyte colony-stimulating factor, Supportive therapy

## Abstract

**Purpose:**

Data indicate that the use of prophylactic granulocyte colony-stimulating factors (G-CSFs) for chemotherapy-induced febrile neutropenia (FN) in routine practice is not consistent with guideline recommendations. The initiative “supportive care for febrile neutropenia prevention and appropriateness of G-CFS use” was undertaken to address the issue of inappropriate prescription of G-CSFs and to improve guideline adherence in the treatment of FN.

**Methods:**

In a two-round Delphi procedure, 36 medical oncologists reviewed clinically relevant recommendations on risk assessment, the appropriate use of G-CSFs, and the prevention of FN based on available literature and individual clinical expertise.

**Results:**

The consensus was reached on 16 out of 38 recommendations, which are backed by evidence from randomised clinical trials and routine clinical practice. The medical oncologists agreed that the severity of neutropenia depends on patients’ characteristics and chemotherapy intensity, and therefore, the risk of severe neutropenia or FN should be assessed at each chemotherapy cycle so as to initiate prophylaxis with G-CSFs if required. The use of biosimilar G-CSFs, with similar efficacy and safety profiles to the originator biologic, has improved the availability and sustainability of cancer care. The timing of supportive therapy is crucial; for example, long-acting G-CSF should be administered 24–72 h after chemotherapy administration. Each biological agent has a recommended administration dose and duration, and it is important to follow these recommendations to avoid complications associated with under-prophylaxis.

**Conclusion:**

It is hoped that these statements will help to increase adherence to guideline recommendations for appropriate G-CSF use and improve patient care.

**Supplementary Information:**

The online version contains supplementary material available at 10.1007/s00520-022-07430-7.

## Introduction

Neutropenia is the most frequent side effect and a significant clinical problem for patients undergoing chemotherapy with anticancer/myelosuppressive drugs [1, 2]. The Common Toxicity Criteria of the National Cancer Institute defines four grades of neutropenia based on the absolute neutrophil count (ANC): grade 1, ANC ≥ 1.5 to < 2 × 10^9^/L; grade 2, ≥ 1.0 to < 1.5 × 10^9^/L; grade 3, ≥ 0.5 to < 1.0 × 10^9^/L; grade 4, < 0.5 × 10^9^/L [1].

Febrile neutropenia (FN) is the most significant complication of neutropenia constituting an oncological emergency and is defined as the appearance of fever (oral temperature > 38.3 °C or two consecutive readings of > 38.0 °C for 2 h) or clinical signs of sepsis in a patient with a neutrophil count of < 0.5 × 10^9^/L (< 500/mm^3^) or < 1.0 × 10^9^/L (< 1000/mm^3^) prenadir [3, 4]. Since obtaining oral temperature measurements at the peak or every 2 h can be challenging in severely ill patients, a lower oral temperature (38 °C) sustained for 1 h can be considered FN [5]. The incidence of FN varies between 2 and 50% depending on patient-related risk factors (e.g. age, neutropenia, major comorbidities, liver or kidney dysfunction, poor performance status, other concurrent immunosuppression or other reasons for compromised bone marrow function), cancer type, chemotherapy regimen (e.g. planned full dose intensity > 85%), and genetic susceptibility, which also influences its clinical outcome [1, 3, 6, 7]. While most patients experience mild episodes, the rate of serious complications (25–30%) and mortality (9–12%) remains elevated in high-risk groups [3]. Furthermore, FN-related mortality is higher in patients requiring intensive care unit-level therapy and in those who develop pneumonia [8, 9]. Owing to the heterogeneity of FN, various decision-making models have been established for the management of patients at the onset of FN. Management options include the prophylactic use of granulocyte colony-stimulating factors (G-CSFs) and the selective use of prophylactic antimicrobial agents [1, 10].

G-CSFs are growth factors that regulate the growth and differentiation of cells in the myeloid lineage [6]. Supportive use of G-CSFs has been shown to reduce the incidence and severity of FN in patients receiving myelosuppressive chemotherapy [6], and is recommended for specific patients in guidelines developed by the American Society of Clinical Oncology (ASCO), the European Organisation for Research and Treatment of Cancer (EORTC), Italian Association of Medical Oncology (AIOM), European Society For Medical Oncology (ESMO), and the National Comprehensive Cancer Network (NCCN) [2–4, 11].

Four recombinant G-CSF formulations are currently in use: filgrastim (nonglycosylated), pegfilgrastim (pegylated filgrastim), lenograstim (glycosylated), and lipefilgrastim (glycopegylated filgrastim). Long-acting pegfilgrastim is created by the covalent attachment of a polyethylene glycol molecule to filgrastim. Pegylation alters the mode of clearance from renal clearance to a self-regulating, neutrophil-mediated mechanism. As a result, pegylated filgrastim has a much longer plasma half-life (15–80 h) than the nonpegylated version (3–4 h), and allows a single administration of the drug per chemotherapy cycle [2, 12].

Although the management and prevention of FN is an integral part of supportive care for many patients undergoing chemotherapy, the originator biological agents filgrastim and pegfilgrastim are costly, which may limit access to these treatments. This limitation can be overcome with biosimilars, which are biological products highly similar to the approved originator [6]. Unfortunately, adherence to treatment guidelines for FN is poor in most places, and the use of non-guideline-based treatments (such as vancomycin) is high [13]. Moreover, non-adherence to applicable FN guidelines increases unnecessary hospital admissions of low-risk patients and frequent over-prescription of empirical antibiotics [14]. One of the factors that may lead to inconsistency between guideline recommendations and routine practice is the fact that FN risk estimation is mainly based on the physician’s experience [15]. Risk estimation may be particularly difficult in vulnerable patients (e.g. elderly) because the evidence is limited in these groups as most randomised clinical trials (RCTs) exclude high-risk individuals [15].

To assist clinicians in risk assessment, the appropriate use of G-CSFs and the prevention of FN, a Delphi consensus process was undertaken to develop clinically relevant recommendations. The current consensus statements address the clinical impact of FN on patient management and the safety and efficacy of G-CSFs and their dosing regimens to increase awareness among clinicians.

## Methods

### Design

The initiative “supportive care for febrile neutropenia prevention and appropriateness of G-CSF use” was undertaken to address the issue of inappropriate prescription of growth factors and disregard of clinical guidelines in the treatment of FN. The current availability of both short-acting filgrastim and long-acting pegfilgrastim, including biosimilars, prompted the meeting of a scientific board of expert Italian medical oncologists (the authors of this paper) to define the most appropriate use of these agents.

This initiative aimed to develop a series of statements on the prevention of FN and the most appropriate use of G-CSFs and arrive at a consensus using the Delphi method, which is an iterative technique focused on reaching consensus among a panel of experts during several rounds of questioning [16].

### Development of consensus statements

#### Preparatory phase

The Delphi process was conducted between July 2020 and July 2021 (Supplementary Figure S1). It began with the meeting of the scientific board in July 2020, where the participants defined the objectives and the topics to be addressed using PICO (population, intervention, comparison, and outcome) questions. The following six topics of interest were identified around which statements and questions were drafted: (1) Clinical impact of FN on patient management (when it affects treatment choices); (2) Awareness of differences between short- and long-acting formulations; (3) Febrile/nonfebrile neutropenia; (4) Timing of the use of long- and short-acting formulations; (5) Toxicity of long- and short-acting agents; 6) Sphere of application of short- or long-acting agents (treatment setting, type of regimen, etc.). Systematic literature searches were performed to prepare preliminary statements supported by published evidence that answered the PICO questions (discussed in detail in Supplementary Methods). The scientific board discussed the statements, based on available literature and personal clinical expertise, about particularly controversial topics on risk assessment, the appropriate use of G-CSFs, and the prevention of FN.

#### Round 1

In the second meeting, convened in September 2020, the scientific board reviewed and finalised the draft of the statements and items to be included in the Delphi questionnaire. Of the 52 questions in the first draft, a 37-item questionnaire was prepared and sent to a panel of 36 medical oncologists (representing different hospitals, universities, and treatment centres across northern, central, and southern Italy; Appendix) for the survey. Each oncologist was asked to grade their agreement with each statement using a 5-point Likert scale (1: strongly disagree; 2: disagree; 3: partially agree; 4: agree; 5: strongly agree).

#### Round 2

In the third meeting convened in May 2021, the scientific board discussed the results of the first round of the Delphi questionnaire. The survey results prompted a re-evaluation of some items, which were modified, reformulated, deleted or added, and the modified statements were sent to the same 36-member panel for another round of voting.

#### Final phase

The fourth and final meeting was conducted in July 2021 to discuss the results of the second round of the Delphi questionnaire. The focus of the scientific board in this meeting was on the items that had been modified after round 1 and for which the degree of consensus had changed.

### Data analysis

The consensus was defined as ≥ 66.6% of participants agreeing/strongly agreeing (scores of 4 or 5). The stability of consensus for all relevant items, i.e. those items that remained unmodified between rounds, was considered reached when the median response remained ≥ 4. The results were validated using the “test of the median for independent samples” from SPSS Statistics software (version 25), which established whether the medians of the first and second rounds were comparable, using a significance level of 0.05%.

## Results and discussion

The Delphi consensus process resulted in a total of 37 recommendations in the first round and 38 in the second round over the six topics (Tables 1, 2, 3, 4, 5, and 6). Thirty-six medical oncologists provided their opinion about the supportive therapies for the prevention of FN and the appropriate use of G-CSFs in round 1 and reached a consensus on 11 out of 37 items. Since six questions were reformulated and one question was added after round 1, a second round was conducted to gauge agreement with the revised statements. Thirty-four of the 36 medical oncologists (94%) who participated in round 1 provided their opinion in round 2, and consensus was reached on 16 out of 38 items.

**Table 1:**
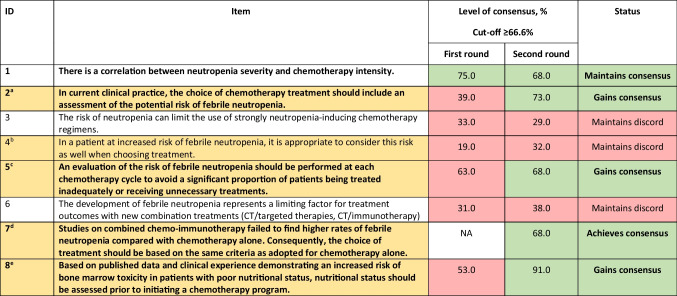
Statements for the clinical impact of febrile neutropenia on patient management (when it affects treatment choices). Statements in bold are those with consensus achieved or maintained at the second round. Statements agreed upon or not agreed upon are colour-coded green and red, respectively. Statements modified between rounds 1 and 2 are colour-coded yellow, and the original statements are given as a footnote

^a^In current clinical practice, the choice of chemotherapy treatment should include an assessment of the potential risk of febrile neutropenia. ^b^In a patient at increased risk of febrile neutropenia, it is appropriate to consider this risk when choosing treatment. ^c^An evaluation of the risk of febrile neutropenia should be performed at each chemotherapy cycle. ^d^The question was not included in round 1 of the Delphi process. ^e^Nutritional status should be assessed prior to initiating a chemotherapy program in order to avoid the risk of bone marrow toxicity

*CT*, chemotherapy; *NA*, not available

**Table 2:**
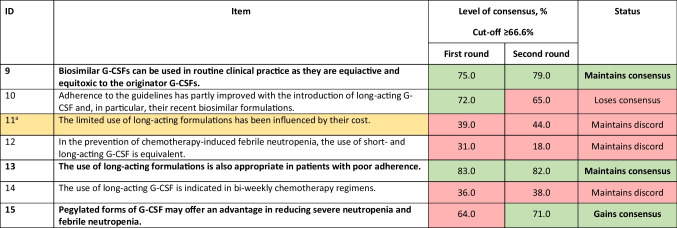
Statements for awareness of differences between short- and long-acting formulations. Statements in bold are those with consensus achieved or maintained at the second round. Statements agreed upon or not agreed upon are colour-coded green and red, respectively. Statement modified between rounds 1 and 2 is colour-coded yellow, and the original statement is given as a footnote

^a^The limited use of long-acting formulations has been influenced by their cost

*G-CSF*, granulocyte colony-stimulating factor

**Table 3:**
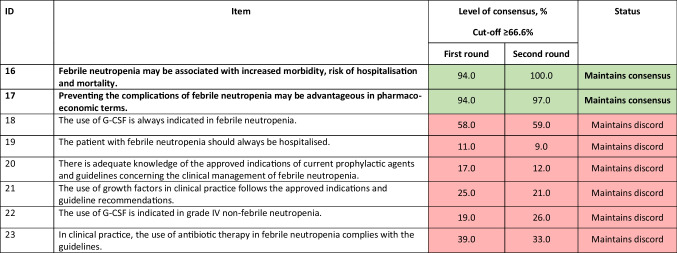
Statements for febrile/nonfebrile neutropenia. Statements in bold are those with consensus achieved or maintained at the second round. Statements agreed upon or not agreed upon are colour-coded green and red, respectively

*G-CSF*, granulocyte colony-stimulating factor

**Table 4:**
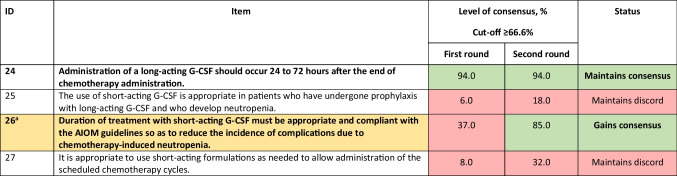
Statements for timing of the use of long- and short-acting formulations. Statements in bold are those with consensus achieved or maintained at the second round. Statements agreed upon or not agreed upon are colour-coded green and red, respectively. Statement modified between rounds 1 and 2 is colour-coded yellow, and the original statement is given as a footnote

^a^Duration of treatment with short-acting G-CSF affects the incidence of complications due to chemotherapy-induced neutropenia

*AIOM*, Italian Association of Medical Oncology; *G-CSF*, granulocyte colony-stimulating factor

**Table 5:**
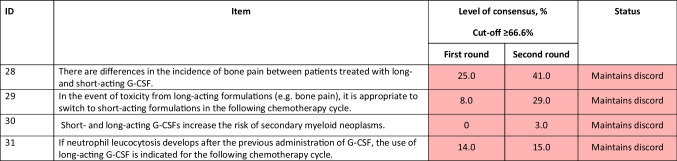
Statements for toxicity of long- and short-acting formulations. Statements agreed upon or not agreed upon are colour-coded green and red, respectively

*G-CSF*, granulocyte colony-stimulating factor

**Table 6:**
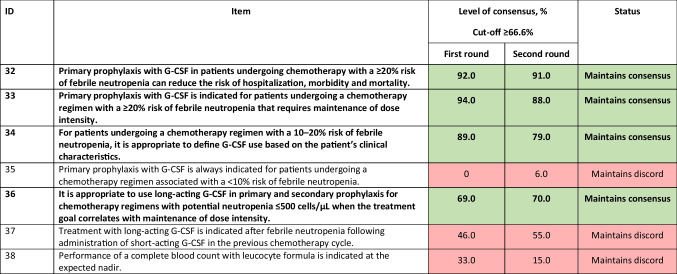
Statements for sphere of application of short- or long-acting agents (treatment setting, type of regimen, etc.). Statements in bold are those with consensus achieved or maintained at the second round. Statements agreed upon or not agreed upon are colour-coded green and red, respectively

*G-CSF*, granulocyte colony-stimulating factor

In the following sections, the consensus statements from each topic will be discussed, along with the most relevant results from the preliminary survey and the supporting scientific evidence when available. The statements which failed to gain consensus are discussed in the Supplementary information (online resource).

### Clinical impact of FN on patient management (when it affects treatment choices)

The consensus was achieved for five of the eight statements on the clinical impact of FN on patient management (Table 1). It was agreed that the severity of neutropenia was dependent on the intensity of chemotherapy (item 1) and that the chemotherapy regimen should be based on the patient’s clinical characteristics and treatment goals after the evaluation of the risk of FN to ensure appropriate treatment (items 2 and 5).

Neutropenia is a dose-limiting side effect of chemotherapy and there is adequate evidence that the severity of neutropenia is dependent on the intensity of the chemotherapy regimen (defined by the type, dose, and number of myelosuppressive cytotoxic agents in a chemotherapy regimen) [1, 15]. A highly intensive chemotherapy regimen is expected to cause more severe myelosuppression, resulting in a higher incidence of FN [17].

Chemotherapy dose and schedule are important clinical variables that can impact patient outcomes, but myelosuppression (mostly FN) drives chemotherapy dose reduction and dose delays. For example, grade 4 neutropenia can result in a 5- to 7-day delay in the next chemotherapy cycle [15, 18]. The incidence of grade 4 neutropenia, or FN, in patients with endometrial cancer was found to be significantly higher with six cycles of chemotherapy compared with four cycles, indicating that patients are likely to benefit from lower haematological toxicity with a shorter duration of chemotherapy [19]. Moreover, patients who develop grade 3 or 4 neutropenia during chemotherapy are at increased risk for developing infections and sepsis, which may lead to dose interruptions or dose reductions and may compromise treatment efficacy [20].

Quantification of the risk associated with patient-related factors and neutropenic events is essential for clinical decision-making as it leads to better clinical outcomes, fewer complications, and closer adherence to treatment protocols [21]. It also prevents patients from being inadequately or unnecessarily treated [22]. Current guidelines suggest assessing FN risk at the start of each chemotherapy cycle in order to prevent or better manage the condition if it arises. A physiological model of granulopoiesis and its regulation has been used to predict ANC time profiles and incidences of neutropenia for chemotherapeutic agents like paclitaxel, carboplatin, gemcitabine, and doxorubicin by incorporating their mechanisms of action. This model can successfully predict individual patient ANC time profiles, which in most patient is a nadir between days 7–14 after the first dose and recovery to grade 2 neutropenia level at the end of cycle one, and could be useful for selecting appropriate therapeutic as well as support strategies for each patient [20].

There was consensus that the incidence of FN was not increased when chemotherapy was combined with immunotherapy (compared with chemotherapy alone); therefore, physicians should use the same criteria to decide on chemo-immunotherapy as they would for chemotherapy (item 7). For instance, FN was the most common grade 3 treatment-related adverse event (AE) when nivolumab was added to platinum-based neoadjuvant chemotherapy in patients with resectable stage IIIA non-small-cell lung cancer, but there was no difference in disease outcome between patients who developed grade 2 to 4 AEs compared with those who developed grade 1 AEs [23].

The Delphi group also agreed that a patient’s nutritional status should be assessed prior to initiating a chemotherapy program, considering that poor nutritional status increases the risk of bone marrow toxicity (item 8). There are a number of studies that support the correlation between poor nutritional status and the risk of high-grade neutropenia [24, 25]. Although obese patients are not at increased risk of FN, they may have a lower threshold for FN and require more antibiotics after chemotherapy [26]. Reportedly, sarcopenic obesity, an independent indicator of poor prognosis in pancreatic cancer patients, also increases the risk of high-grade neutropenia [27]. According to a meta-analysis of RCTs, neutropenic diets do not reduce the risk of FN [28].

One approach to reducing the risk of FN in patients with neutropenia is to modify the chemotherapy protocol. The eviQ website provides an online resource with treatment protocols and recommendations for dose modification (due to haematological toxicity). However, a survey of Medical Oncology Group of Australia (MOGA) members and eviQ reference committee members indicated that the majority of the medical oncologists do not follow dose modification guidelines as they consider them to be too conservative [29]. An alternative approach then is to use G-CSF rather than changing the dose intensity of the chemotherapeutic agents [15].

### Awareness of differences between short- and long-acting formulations of filgrastim

Although the strategies for decreasing the risk of FN and its complications mostly include chemotherapy dose reductions and delays, prophylactic use of G-CSFs has markedly reduced the incidence of FN and related comorbidities [15, 30]. A meta-analysis of RCTs showed that the G-CSF treatment significantly reduces the time spent in the hospital and time to neutrophil recovery but does not significantly change overall mortality or infection-related mortality [31]. However, this study was not statistically powered to assess mortality [31]. A systemic review and meta-analysis of RCTs comparing chemotherapy with or without primary prophylaxis with G-CSF showed a significant reduction in all-cause mortality with G-CSF therapy, particularly in patients receiving dose-dense chemotherapy [32].

Biosimilar versions of G-CSFs that have similar pharmacodynamic and pharmacokinetic profiles to the originator biological agent have helped to improve access to supportive cancer care and the sustainability of cancer treatment [33]. The panel reached a consensus on the use of biosimilar G-CSFs in routine clinical practice (Table 2) as they agreed that the activity and toxicity profiles of biosimilar G-CSFs were comparable with those of the original G-CSFs (item 9). A number of studies comparing the efficacy and safety of the US Food and Drug Administration (FDA) and European Medicines Agency–approved originator filgrastim and its various biosimilars found them to be highly similar with respect to primary, secondary, and tertiary protein structures, as well as mass, size, purity, charge, and hydrophobicity. There was no difference in receptor binding affinity nor in vitro bioactivity [34, 35]. Similarly, no meaningful differences in safety, local tolerability, or immunogenicity were identified between biosimilar pegfilgrastim and the originator biologicals, establishing their bioequivalence [33, 36].

They also agreed that long-acting formulations of G-CSFs are effective in patients with poor adherence (item 13) and offer an advantage in reducing severe neutropenia and FN (item 15). Contrary to the daily administration of filgrastim (5 μg/kg; short-acting G-CSF) until post-nadir ANC recovery to near normal levels (which may take up to 14 days), pegfilgrastim is administered as a single subcutaneous injection (6 mg) once after each chemotherapy cycle, resulting in fewer injections, fewer hospital visits, and better patient adherence [6, 37, 38]. A study by Almenar et al. found that, compared with short-acting G-CSF, primary prophylaxis with long*-*acting G-CSF provided greater protection against grade 3 and 4 neutropenia and FN (odds ratio [OR] 3.1, 95% confidence interval [CI]: 1.1–8.8) and was associated with fewer chemotherapy dose delays and reductions and a higher response rate (OR 2.1, 95%CI: 1.2–3.7) [39]. Another study by Pinto et al. found that a single dose of long-acting G-CSF performed better than a median of 10–14 days of short-acting G-CSF in reducing FN rates for patients undergoing myelosuppressive chemotherapy [40]. In addition, a review of real-world comparative effectiveness studies suggested that the risks of FN and FN-related complications were generally lower for prophylaxis with long-acting versus short-acting G-CSFs [41].

### Febrile/nonfebrile neutropenia

Despite medical advances, neutropenia (both febrile and nonfebrile) is still considered an oncological emergency, associated with considerable morbidity, mortality, and costs [15]. Therefore, it was no surprise that the expert panel reached a consensus (Table 3) on the association of FN with increased morbidity, mortality, and risk of hospitalisation (item 16) and that the management of FN offers pharmacoeconomic advantages (item 17). Many studies have established the association between chemotherapy-induced neutropenia and an increased risk of morbidity, mortality, and hospitalisation, with estimates of 6.8 to 20% mortality among patients who are hospitalised for FN-related complications and with higher rates observed in patients who have major comorbidities and documented sepsis or shock [10, 21].

To reduce costs while improving disease outcomes, we need evidence-based surveillance after curative therapy, a reduction in the unnecessary use of G-CSFs, better integration of palliative care into usual oncology care, and the use of evidence-based, cost-conscious clinical pathways that would lead to better outcomes at one-third lower cost [42]. Appropriate G-CSF administration is associated with a decrease in complications (especially infections and sepsis) and a consequent decrease in neutropenia duration, faster recovery from fever, and reduced hospital stay, leading to an overall reduction in treatment cost [43]. A Belgian study showed that primary prophylaxis of FN with pegfilgrastim is cost-effective compared with other prophylactic strategies in patients with stage II breast cancer or non-Hodgkin lymphoma at a threshold of €30,000/QALY (quality-adjusted life-year) [44].

### Timing of the use of long- and short-acting formulations

The timing of supportive therapy is crucial for patient management. As shown in Table 4, the consensus was achieved on the administration of a long-acting G-CSF 24 to 72 h after chemotherapy administration (item 24). The panel also agreed that there is a correlation between the duration of treatment with short-acting G-CSFs and complications associated with chemotherapy-induced neutropenia (item 26).

In a large-scale evaluation of > 45,000 adults who received intermediate/high-risk regimens for solid tumours or non-Hodgkin lymphoma, FN incidence was significantly higher among those who received pegfilgrastim prophylaxis on the same day as chemotherapy completion versus 24–72 h after chemotherapy completion [45], as is recommended in the guidelines. Initiation of G-CSF prophylaxis is recommended at 24 h after completion of chemotherapy because the rapidly dividing myeloid progenitor cells induced by G-CSF might be sensitive to residual cytotoxic agents, which increases the risk of FN [6, 45]. However, G-CSF should be administered within 72 h of chemotherapy when the bone marrow is still regenerative and able to respond to treatment [46].

However, some evidence suggests that G-CSF initiated < 24 h may benefit some patients [3, 6, 47, 48]. For example, real-world evidence from the MONITOR-GCSF study, in which 92% of the patients had solid tumours, nearly half were aged > 65 years, and 57% received chemotherapy as adjuvant treatment, indicated that the risk of chemotherapy-induced neutropenia was similar between patients who received biosimilar filgrastim on the same-day as chemotherapy (< 24 h) and those who received it 24–74 h post-chemotherapy. These data indicate that same day G-CSF prophylaxis may be appropriate in a select subgroup of patients and is subject to clinicians’ judgment and patient preferences and barriers [48].

There is a correlation between the duration of treatment with short-acting G-CSFs and complications associated with chemotherapy-induced neutropenia. A shorter duration of prophylaxis with short-acting G-CSF was found to increase the risk of FN and lead to worse neutropenia-related clinical outcomes [49].

Another study comparing the use of pegfilgrastim in patients with gynaecological cancers on the same day as a myelosuppressive chemotherapy regimen (day 1) compared with the day after (day 2) showed that day 1 administration was less costly (US $17,195 versus US $17,681) and resulted in a better quality of life than day 2 administration (0.2298 QALYs versus 0.2288 QALYs), possibly due to reduced treatment visits [50]. While these findings suggest that same-day administration of pegfilgrastim may have some benefits, further research is needed before a change to guideline-based practice can be recommended.

### Toxicity of long- and short-acting formulations

Bone pain is the commonly reported AE associated with G-CSF use, ranging from 25 to 38%. First-line treatment for bone pain involves acetaminophen and nonsteroidal anti-inflammatory agents (e.g. naproxen), while antihistamines (e.g. loratadine), opioids, and dose reduction of G-CSFs are considered second-line therapy [51, 52]. Apart from bone pain, there are also reports of G-CSF-associated vasculitis that may be accompanied by severe complications like aortic dissection and aneurysm formation [53]. A G-CSF-associated vasculitis is a rare event, with an incidence of 0.5%, and usually involves large vessels like the thoracic and abdominal aortae, and brachiocephalic, subclavian, common carotid, and temporal arteries [53]. None of the statements on the toxicity of the short- and long-acting formulations achieved consensus (items 28 to 31; Table 5).

### Sphere of application of short- or long-acting agents

According to the consensus (Table 6), primary prophylaxis with G-CSF should be considered in patients with a ≥ 20% risk of chemotherapy-induced FN to reduce FN-related complications and to avoid chemotherapy dose reduction (items 32 and 33), as well as in patients with 10–20% risk of developing FN, depending on the patient’s clinical characteristics (item 34). The panel also agreed that it is appropriate to use long-acting G-CSF in primary and secondary prophylaxis during chemotherapy regimens with the potential to induce neutropenia of ≤ 500 cells/μL when the treatment goal correlates with maintenance of dose intensity (item 36).

In this respect, the consensus recommendations are consistent with the ASCO, ESMO, NCCN, AIOM, Spanish Society of Medical Oncology (SEOM), and EORTC guidelines for primary prophylaxis in chemotherapy regimens, which recommend G-CSF as primary prophylaxis in patients receiving chemotherapy in the following circumstances: when the likelihood of developing FN is > 20%, when there is an intermediate risk (10–20%), but the risk of FN is increased (patient aged > 65 years, advanced disease, poor performance status, liver or kidney dysfunction, recent extensive surgery, persistent neutropenia, prior episodes of FN, poor nutritional status, widespread bone marrow involvement, multimorbidity, or frailty), or when the consequences of the neutropenic episode are foreseen to be more severe [2–4, 11]. These guidelines do not recommend G-CSF as primary prophylaxis in patients with < 10% risk of FN or as secondary prophylaxis in patients with a previous episode of FN (in a previous cycle of chemotherapy), where a dose reduction is not recommended as it may affect overall survival or disease-free survival. There are limited data on real-world outcomes of G-CSF prophylaxis in patients with < 10% risk of FN and very little information about the most vulnerable patients, their risk factors for FN or serious FN outcomes.

## Conclusion

The results of this Delphi study have provided recommendations in several areas of the management of FN using G-CSFs and provided guidance on the safety, efficacy, and cost-effectiveness of short- and long-acting G-CSFs. The Delphi method proved to be an appropriate way to compile treatment recommendations in the field of FN prophylaxis and treatment, which has been plagued by poor adherence to guidelines and inadequate use of G-CSFs in recent years. It is hoped that these statements will help to increase adherence to guideline recommendations and improve patient outcomes.

### Electronic supplementary material

Below is the link to the electronic supplementary material.

Supplementary file1 (DOCX 84 kb)

## Data Availability

The datasets analysed in this study are available from the corresponding author upon reasonable request.

## Appendix

Medical oncologists representing different hospitals, universities and treatment centres across northern, central and southern Italy (*n* = 36).

**Table:**

Delphi panel members (medical oncologists)	Affiliations
Northern Italy	
Ilaria Attili	IRCCS, European Institute of Oncology, Division of Thoracic Oncology, Milan
Alfredo Berruti	Azienda Ospedaliera Spedali Civili di Brescia, Università degli Studi di Brescia Dipartimento di Specialità Medico-Chirurgiche, Scienze Radiologiche e Sanità Pubblica, Brescia
Lucia Bonomi	ASST-Ospedale Papa Giovanni XXIII Bergamo, USC Oncologia, Bergamo
Silvia Bozzarelli	IRCCS Humanitas Research Hospital, Humanitas Cancer Center Medical Oncology and Hematology Unit, Rozzano, Milan
Diego Luigi Cortinovis	ASST H. S. Gerardo, SC Oncologia Medica/SS Lung Unit, Monza
Francesco Grossi	Università degli Studi dell’Insubria, UOC Oncologia Medica, Varese
Matteo Lambertini	IRCCS Ospedale Policlinico San Martino, Department of Medical Oncology, Clinica di Oncologia Medica, Genova; University of Genova, Department of Internal Medicine and Medical Specialties (DiMI), School of Medicine, Genova
Manlio Mencoboni	Ospedale Villa Scassi, Asl 3 Genovese, SSD Oncologia, Genova
Fulvia Pedani	Azienda Ospedaliera della Salute e della Scienza di Torino, Coordinamento Ambulatorio- DayHospital S.C. Oncologia Medica 2, Torino
Rebecca Pedersini	ASST Spedali Civili, Brescia, Breast Unit-Oncology, Brescia
Emma Pozzi	Ospedale civile di Voghera, ASST Pavia, Unità Operativa Semplice Oncologia, Pavia
Francesco Raspagliesi	Fondazione IRCCS Istituto Nazionale Tumori, Milano
Alessia Rognone	IRCCS Ospedale San Raffaele, Medical Oncology Department, Milano
Lorenzo Sica	IRCCS Ospedale San Raffaele, U.O. Oncologia Medica, Milano
Andrea Sponghini	A.O.U. Maggiore della carità Novara, SCDU Oncologia, Novara
Silvia Stragliotto	Istituto Oncologico Veneto IOV – IRCCS, Dipartimento di Oncologia UOC Oncologia 3, Castelfranco Veneto (TV)
Emiliana Tarenzi	Grande Ospedale Metropolitano Niguarda, Niguarda Cancer Center, Oncologia Falck, Dipartimento Ematologia, Oncologia e Medicina Molecolare, Milano
Central Italy	
Andrea Antonuzzo	Azienda Ospedaliera Universitaria Pisana, U.O. Polo Oncologico, U.O. Oncologia Medica 1 SSN, Pisa
Beatrice Detti	Azienda Ospedaliero-Universitaria Careggi, Radioterapia Oncologica, Firenze
Francesco Di Clemente	Azienda usl Toscana sud est, UOSD Oncologia Medica Valdarno, Montevarchi (AR)
Paola Fuso	Fondazione Policlinico Universitario Agostino Gemelli I.R.C.C.S., Department of Woman and Child Health and Public Health, Roma Italy; Università Cattolica Del Sacro Cuore, Faculty of Medicine and Surgery, Roma
Alain J. Gelibter	Policlinico Umberto I Roma, UOC Oncologia B, Roma
Mario Roselli	Università degli Studi di Roma Tor Vergata, Dipartimento di Medicina dei Sistemi, Roma
Vieri Scotti	Azienda Ospedaliero-Universitaria Careggi, Radioterapia oncologica, dipartimento oncologia, Firenze
Southern Italy	
Raffaele Ardito	IRCCS Centro di Riferimento Oncologico della Basilicata—(CROB), Rionero in Vulture (Pz)
Roberta Caputo	Istituto Nazionale Tumori IRCCS “Fondazione G. Pascale”, Breast Unit, Napoli
Carmine De Angelis	Università degli Studi Napoli “Federico II”, Dipartimento di Medicina Clinica e Chirurgia, Napoli
Piergiorgio Di Tullio	Policlinico Riuniti di Foggia, Oncologia Medica e Terapia Biomolecolare, Foggia
Francesco Giotta	I.R.C.C.S. Istituto Tumori “Giovanni Paolo II” di Bari, Unità Operativa Complessa di Oncologia Medica, Bari
Antonio Mafodda	Grande Ospedale Metropolitano, Oncologia Medica, Reggio Calabria
Michele Montrone	I.R.C.C.S. Istituto Tumori "Giovanni Paolo II" di Bari, Thoracic Oncology Unit, Bari
Giuliano Palumbo	Istituto Nazionale Tumori I.R.C.C.S. “Fondazione G. Pascale” di Napoli, Oncologia Medica Toraco Polmonare, Napoli
Livio Puglia	AORN—Azienda Ospedaliera Antonio Cardarelli, Napoli
Clementina Savastano	A.O.U. San Giovanni di Dio e Ruggi d’Aragona, Salerno
Salvatore Tafuto	Istituto Nazionale Tumori IRCCS " Fondazione G. Pascale", Direttore f.f.S.C. Sarcomi e turmori rari, Napoli
Salvatore Turano	Azienda Ospedaliera di Cosenza, UOC Oncologia, Cosenza
