# Supplementary material for: Supportive therapies in the prevention of chemotherapy-induced febrile neutropenia and appropriate use of granulocyte colony-stimulating factors: a Delphi consensus statement
Source: Support Care Cancer. 2022 Nov 5;30(12):9877–88. doi: 10.1007/s00520-022-07430-7 (PMC9715510; doi:10.1007/s00520-022-07430-7)
Supplement: Supplementary file 1 — Supplementary file1 (DOCX 84 kb) [file 520_2022_7430_MOESM1_ESM.docx]

***Supportive therapies in the prevention of chemotherapy-induced*** ***febrile neutropenia and appropriate use of granulocyte colony-stimulating factors:***

***a Delphi consensus statement***

**Journal**

Supportive Care in Cancer

**Authors**

Vincenzo Adamo, Lorenzo Antonuzzo, Marco Danova, Michelino De Laurentis, Paolo Marchetti, Carmine Pinto, Giovanni Rosti

**Corresponding author:**

Giovanni Rosti

Fondazione IRCCS Policlinico San Matteo,

Viale Camillo Golgi, 19, 27100 Pavia PV, Italy.

Email: [rosti.giovanni@gmail.com](mailto:rosti.giovanni@gmail.com)

# Supplementary Material

## Supplementary methods

### Literature search

We conducted a systematic literature search of PubMed, Medline, Scientific Societies websites and guideline databases between October and November 2020 using the search terms based on the PICO (population, intervention, comparison, outcome) questions identified by the expert panel of the Italian medical oncologists (Supplementary Table S1). Guidelines, systematic reviews, meta-analyses and consensus statements in English and Italian languages were chosen based on the title and abstract. **Supplementary Table S1.** Search terms used in PubMed database.

| **MeSH terms** | **Free text in [Text Word] Field** |
| --- | --- |
| "Febrile Neutropenia"[Mesh] OR "Neutropenia/chemically induced"[Mesh] OR "Chemotherapy-Induced Febrile Neutropenia"[Mesh] OR "Leukocyte Count"[Mesh] OR "Antineoplastic Agents"[Mesh]  "Chemotherapy OR Adjuvant"[Mesh] OR  "Mortality"[Mesh] OR "mortality" [Subheading] OR "Hospital Mortality"[Mesh] OR "Morbidity"[Mesh] OR "Epidemiology"[Mesh] OR "Hospitalization"[Mesh] OR "Length of Stay"[Mesh] | neutropenia*[Text Word] OR neutropaenia*[Text Word] OR neutrop*[Text Word] OR "chemotherapy induced febrile neutropaenia"[Text Word] OR "chemotherapy induced febrile neutropenia"[Text Word] OR "chemotherapy-induced febrile neutropaenia"[Text Word] OR "chemotherapy-induced febrile neutropenia"[Text Word] OR "febrile neutropaenia"[Text Word] OR "febrile neutropenia"[Text Word] OR "neutropenic fever"[Text Word] OR "neutropaenic fever"[Text Word] OR "neutropenic febrile"[Text Word] OR (neutrop*[Text Word] OR fever[Text Word]) OR (neutrop*[Text Word] OR febrile[Text Word]) OR (neutrop*[Text Word] OR chemother*[Text Word]) OR (cancer*[Text Word] OR oncol*[Text Word] OR tumor*[Text Word] OR tumour*[Text Word] OR neoplasm*[Text Word] OR carcinoma*[Text Word]) hemogram*[Text Word] OR haemogram*[Text Word] OR leukocyt*[Text Word] OR hemogram*[Text Word] OR  chemother*[Text Word] OR antineoplas*[Text Word] OR anticancer*[Text Word] OR anticarcinoma*[Text Word] OR antitumor*[Text Word] OR anti-neoplas*[Text Word] OR anti-cancer*[Text Word] OR anti-carcinoma*[Text Word] OR anti-tumor*[Text Word] OR anti-tumour*[Text Word] OR morbilit*[Text Word] OR mortalit*[Text Word] OR hospitalization[Text Word] OR hospitalisation[Text Word] OR febrile[Text Word] OR fever[Text Word] OR neutropen*[Text Word] OR neutropaen*[Text Word] OR mortalit*[Text Word] OR morbilit*[Text Word] OR hospitalisation*[Text Word] OR hospitalization*[Text Word] OR febrile[Text Word] OR fever[Text Word] OR neutropen*[Text Word] OR neutropaen*[Text Word] OR mortalit*[Text Word] OR morbilit*[Text Word] OR comorbilit*[Text Word] OR morbidit*[Text Word] OR comorbidit*[Text Word] OR hospitalisation*[Text Word] OR hospitalization*[Text Word] OR febrile[Text Word] OR fever[Text Word] OR neutropen*[Text Word] OR neutropaen*[Text Word] OR mortalit*[Text Word] OR morbilit*[Text Word] OR comorbilit*[Text Word] OR morbidit*[Text Word] OR comorbidit*[Text Word] OR hospitalisation*[Text Word] OR hospitalization*[Text Word] OR "length of stay"[Text Word] OR length-of-stay[Text Word] OR febrile[Text Word] OR fever[Text Word] OR neutropen*[Text Word] OR neutropaen*[Text Word] OR mortalit*[Text Word] OR morbilit*[Text Word] OR comorbilit*[Text Word] OR morbidit*[Text Word] OR comorbidit*[Text Word] OR hospitalisation*[Text Word] OR hospitalization*[Text Word] OR "length of stay"[Text Word] OR "length-of-stay"[Text Word] OR epidemiol*[Text Word] |
| "Diet OR Food OR and Nutrition"[Mesh] OR "Nutrition Assessment"[Mesh] OR "Nutritional Status"[Mesh] OR "Malnutrition"[Mesh] OR "Obesity"[Mesh] OR "Obesity OR Abdominal"[Mesh] OR "Obesity OR Morbid"[Mesh] OR "Body Mass Index"[Mesh] OR "Overweight"[Mesh] OR “Bone”[MeSH] OR “Pain”[MeSH] | nutrition*[Text Word] OR obes*[Text Word] OR sarcopen*[Text Word] OR malnutrit*[Text Word] OR diet*[Text Word] OR overweight*[Text Word] OR "Body Mass Index"[Text Word] OR BMI[Text Word] OR pain*[Text Word] OR bone*[Text Word] |
| "pegfilgrastim" [Supplementary Concept] OR "Filgrastim"[Mesh] OR "Granulocyte Colony-Stimulating Factor"[Mesh] OR "Anti-Bacterial Agents"[Mesh] AND "Anti-Bacterial Agents" [Pharmacological Action] | "b 12019"[Text Word] OR b12019[Text Word] OR cavoley[Text Word] OR cegfila[Text Word] OR "chs 1701"[Text Word] OR chs1701[Text Word] OR "ckd 12101"[Text Word] OR ckd12101[Text Word] OR "da 3031"[Text Word] OR da3031[Text Word] OR efgratin[Text Word] OR fulphila[Text Word] OR g-lasta[Text Word] OR "gbs 010"[Text Word] OR gbs010[Text Word] OR grasustek[Text Word] OR "hsp 130"[Text Word] OR hsp130[Text Word] OR "ins 20"[Text Word] OR ins20[Text Word] OR "intp 5"[Text Word] OR intp5[Text Word] OR "krn 125"[Text Word] OR krn125[Text Word] OR "la ep2006"[Text Word] OR laep2006[Text Word] OR LA-EP2006[Text Word] OR lapelga[Text Word] OR lipegfilgrastim[Text Word] OR "lupifil p"[Text Word] OR lupifil-p[Text Word] OR "mk 6302"[Text Word] OR mk6302[Text Word] OR "msb 11455"[Text Word] OR msb11455[Text Word] OR "myl 1401h"[Text Word] OR myl1401h[Text Word] OR neulasta[Text Word] OR neulasta onpro[Text Word] OR neulastrim[Text Word] OR neupeg[Text Word] OR neupopeg[Text Word] OR nyvepria[Text Word] OR "peg filgrastim"[Text Word] OR "peg-filgrastim"[Text Word] OR "peg neutrogen"[Text Word] OR pegasta[Text Word] OR pegcyte[Text Word] OR "pegfilgrastim*"[Text Word] OR "pegylated apo-filgrastim"[Text Word] OR "pegylated filgrastim"[Text Word] OR pelgraz[Text Word] OR pelmeg[Text Word] OR "pf 06881894"[Text Word] OR "pf 529"[Text Word] OR "pf 6881894"[Text Word] OR pf06881894[Text Word] OR pf529[Text Word] OR pf6881894[Text Word] OR "r 1471"[Text Word] OR r1471[Text Word] OR "rgb 02"[Text Word] OR rgb02[Text Word] OR ristempa[Text Word] OR "sd 01"[Text Word] OR sd01*[Text Word] OR sd-01-filgrastim[Text Word] OR tinapeg[Text Word] OR "tpi 120"[Text Word] OR tpi120[Text Word] OR udenyca[Text Word] OR ziextenzo[Text Word] OR zioxtenzo[Text Word] OR PEG-rmetHuG-CSF[Text Word] OR PEG SD-01[Text Word] OR XM22[Text Word] OR XM-22[Text Word] OR accofil[Text Word] OR "ax 200"[Text Word] OR ax200[Text Word] OR biofigran[Text Word] OR biograstim[Text Word] OR "bnt 002"[Text Word] OR bnt002[Text Word] OR colstim[Text Word] OR "da 3030"[Text Word] OR da3030[Text Word] OR "ep 2006"[Text Word] OR ep2006[Text Word] OR fegrast[Text Word] OR ficocyte[Text Word] OR filgrastim*[Text Word] OR gracin[Text Word] OR grafeel[Text Word] OR gran[Text Word] OR granix[Text Word] OR granulen[Text Word] OR granulokine[Text Word] OR granulostim[Text Word] OR grasin[Text Word] OR grastofil[Text Word] OR grimatin[Text Word] OR "ins 19"[Text Word] OR ins19[Text Word] OR "krn 8601"[Text Word] OR krn8601[Text Word] OR leucostim[Text Word] OR leukostim[Text Word] OR meograstim[Text Word] OR "mk 4214"[Text Word] OR mk4214[Text Word] OR "mngx 100"[Text Word] OR mngx100[Text Word] OR neopogen[Text Word] OR neotromax[Text Word] OR neukine[Text Word] OR neupogen[Text Word] OR neutrogen[Text Word] OR neutromax[Text Word] OR neutroval[Text Word] OR nivestim[Text Word] OR nivestym[Text Word] OR nufil[Text Word] OR nupen[Text Word] OR nupogen[Text Word] OR ratiograstim[Text Word] OR religrast[Text Word] OR scilocyte[Text Word] OR "tbo filgrastim"[Text Word] OR tbo-filgrastim[Text Word] OR tevagrastim[Text Word] OR topneuter[Text Word] OR "tx 01"[Text Word] OR tx01[Text Word] OR "xm 02"[Text Word] OR xm02[Text Word] OR zarxio[Text Word] OR zarzio[Text Word] OR "Recombinant-Methionyl Human Granulocyte Colony-Stimulating Factor"[Text Word] OR G-CSF RecombinantOR Human Methionyl[Text Word] OR Recombinant Methionyl Human Granulocyte Colony Stimulating Factor[Text Word] OR Tbo-Filgrastim[Text Word] OR Granix[Text Word] OR topneuter[Text Word] OR neupogen[Text Word] OR R-metHuG-CSF[Text Word] OR "R-metHuG CSF"[Text Word] OR long-acting*"[Text Word] OR short-acting*"[Text Word] OR "granulocyte colony stimulating factor*"[Text Word] OR "g csf"[Text Word] OR gcsf[Text Word] OR G-CSF[Text Word] OR "granulocyte colony stimulating factor*"[Text Word] OR "granulocyte colony stimulating factor"[Text Word] OR "granulocyte colony-stimulating factor*"[Text Word] OR "granulocyte-colony-stimulating factor"[Text Word] |
| "Treatment Adherence and Compliance"[Mesh] OR "Guideline Adherence"[Mesh] OR "Patient Compliance"[Mesh] OR "Medication Adherence"[Mesh] OR | "adherence to therap*"[Text Word] OR "adherence to treatment*"[Text Word] OR "compliance to therapy"[Text Word] OR "compliance to treatment"[Text Word] OR "patient adherence"[Text Word] OR "patients' adherence"[Text Word] OR "therapy adherence"[Text Word] OR "therapy compliance"[Text Word] OR "treatment adherence"[Text Word] OR "treatment adherence"[Text Word] OR compliance[Text Word] OR "dosage adherence"[Text Word] OR "dosage compliance"[Text Word] OR "dose adherence"[Text Word] OR "dose compliance"[Text Word] OR "dosing adherence"[Text Word] OR "dosing compliance"[Text Word] OR "drug adherence"[Text Word] OR "drug compliance"[Text Word] OR "drug intake compliance"[Text Word] OR "drug regimen adherence"[Text Word] OR "drug regimen compliance"[Text Word] OR "medication adherence"[Text Word] OR "medication intake adherence"[Text Word] OR adherence[Text Word] |
| "Biosimilar Pharmaceuticals"[Mesh] OR "Therapeutic Equivalency"[Mesh] | biosimilar*[Text Word] OR bio-similar*[Text Word] OR "similar biolog*"[Text Word] OR bioequivalen*[Text Word] OR bio-equivalen*[Text Word] OR equivale*[Text Word] OR equi-valen*[Text Word] |
| "Meta-Analysis"[Publication Type] OR "Meta-Analysis as Topic"[Mesh] OR "Systematic Review" [Publication Type] OR "Systematic Reviews as Topic"[Mesh] OR "Guideline" [Publication Type] OR "Guidelines as Topic"[Mesh] OR "Guideline Adherence"[Mesh] OR "Practice Guideline" [Publication Type] OR "Consensus"[Mesh] OR "Consensus Development Conference OR NIH" [Publication Type] OR "Consensus Development Conference" [Publication Type] OR "Consensus Development Conferences OR NIH as Topic"[Mesh] OR "Consensus Development Conferences as Topic"[Mesh] | meta-analysis[Text Word] OR metanalysis[Text Word] OR metaanalysis[Text Word] OR review*[Text Word] OR "systematic review*"[Text Word] OR consensus[Text Word] OR SR[Text Word] OR "literature review*"[Text Word] OR guideline*[Text Word] |

### Development of consensus statements

**Supplementary Figure S1.** Flow chart of the stages of the Delphi process, including the preparatory phase, the actual Delphi rounds, interim steps of data processing and analysis, and concluding steps.

## Statements failing to achieve consensus

Twenty-two of the 38 statements on the prevention of febrile neutropenia (FN) and the most appropriate use of granulocyte colony-stimulating factors (G-CSFs) did not gain consensus, with discord being maintained for 21 statements between rounds 1 and 2 of the Delphi process. Only one statement lost consensus in round 2. All those statements are listed here under their respective topics, and will be backed by evidence from clinical studies or from routine clinical practice when available.

### Clinical impact of FN on patient management (when it affects treatment choices)

The panel could not reach a consensus on the following three statements for which there are areas of uncertainty in clinical practice (see Table 1 in article): risk of neutropenia is a limiting factor for the use of strongly neutropenia-inducing chemotherapy regimens (item 3), risk of FN dictates the treatment of choice (item 4) and FN development affects treatment outcomes during new combination treatments (such as chemotherapy + targeted therapies or chemotherapy + immunotherapy) (item 6).

### Awareness of differences between short- and long-acting formulations of filgrastim

Four statements failed to gain consensus (see Table 2 in article). They include: availability of long-acting G-CSFs and their biosimilars has increased adherence to treatment guidelines (item 10), the cost of long-acting G-CSFs is the reason for their limited use (item 11), short- and long-acting G-CSFs are equivalent in the prevention of FN (item 12) and long-acting formulations are indicated in bi-weekly chemotherapy regimens (item 14).

The panel initially agreed that the availability of long-acting G-CSFs and their biosimilars has increased adherence to treatment guidelines (item 10), but consensus was lost in round two of voting (72% in round 1 and 65% in round 2). Educational initiatives are imperative as adherence to treatment guidelines is poor in most places and use of non-guideline-based treatments (such as vancomycin) is high [1]. For example, routine administration of G-CSF prophylaxis in Italy is frequently not in accordance with Italian Association of Medical Oncology (AIOM) guidelines and often leads to poor outcomes [2]. Link et al. found that adherence to G-CSF indications improved in Germany after publication of the 2010 European Organisation for Research and Treatment of Cancer (EORTC) guideline update, but they emphasized the need for continuing medical education for oncologists to promote adherence to guidelines on G-CSF administration [3]. Data have shown that expert peer-to-peer consultation with prescribing oncologists can promote adherence to guidelines that will result in significant cost reductions and decreased risk of FN [4]. It is however hypothesized that long-acting G-CSFs may improve clinicians’ adherence to guidelines by providing an acceptable and convenient approach to FN prevention, particularly for elderly and vulnerable patients [5].

Although the panel did not agree that the cost of long-acting G-CSFs is the reason for their limited use (item 11), evidence suggests that the availability of G-CSF biosimilars have led to cost savings compared with the use of the reference product, especially during cost-intensive long-term maintenance treatments and therapies with high FN incidence, and has increased access to G-CSF supportive care [6-9]. The panel also did not agree on the equivalence of short- and long-acting G-CSFs in the prevention of FN (item 12). While randomised studies suggest that there no significant differences in their efficacy or cost effectiveness [10, 11], greater efficacy for long-acting CSFs was found in non-randomised controlled trials, possibly because of the frequent under-dosing of short-acting G-CSFs in real-world routine practice. For example, <3 doses of G-CSF per chemotherapy cycle are used in 45% of cases and four to five doses in 37% of cases in clinical practice in Italy, while 11 doses/cycle were administered in clinical trials [5]. The problem of under-dosing of short-acting G-CSFs can be solved by using long-acting G-CSF in routine clinical practice, and implementing guidelines with multidisciplinary support in the hospital [12].

The panel’s disagreement on the use of long-acting formulations in bi-weekly chemotherapy regimens (item 14) was expected as the US Food and Drug Administration (FDA) label for pegfilgrastim specifies that it should not be administered 14 days before or within 24 hours of administration of myelosuppressive chemotherapy. Thus, prophylactic use of pegfilgrastim with a biweekly chemotherapy regimen is not possible [13]. Notwithstanding, a few studies have supported pegfilgrastim use in biweekly regimens. Santini et al. showed a transient increase in white blood cells and polymorphonuclear leukocytes that settled with the second dose. They believed that pegfilgrastim in biweekly regimens will prevent treatment delays and should be considered [14]. In addition, Draper et al. suggested that administration of pegfilgrastim on the same day as 5-fluorouracil and in biweekly chemotherapy regimens is safe and effective [15].

### Febrile/non-febrile neutropenia

No consensus could be achieved on six out of eight statements (see Table 3 in article). They include: G-CSF is always indicated in FN (item 18) and in grade 4 non-febrile neutropenia (item 22), patients with FN should always be hospitalised (item 19), and there is adequate knowledge about the registration indications and guidelines concerning the clinical management of FN with growth factors and antibiotics (items 20, 21 and 23).

According to Spanish Society of Medical Oncology (SEOM) guidelines, hospitalisation is not necessary for patients who do not meet specific severity criteria based on the presence of vascular, haematological, gastrointestinal, infectious, neurological, pulmonary, or other risk factors [16]. Updated clinical practice guidelines of the American Society of Clinical Oncology (ASCO) and Infectious Diseases Society of America (IDSA) recommend empirical antibacterial therapy with an oral fluoroquinolone plus amoxicillin/clavulanate (or clindamycin, if penicillin allergic) within 1 hour of triage for the outpatient management of patients with FN, who should then be monitored for at least 4 hours before discharge. If there is no abatement of fever after 2–3 days of an empirical broad-spectrum antibiotic regimen, the patients should be re-evaluated and considered for hospitalisation [17]. Guidelines continue to recommend early empiric antimicrobials or G-CSF plus antibiotics in FN, but there are contradictory results on the impact on hospital stay and mortality [18, 19]. Duration of neutropenia in afebrile patients can be reduced with routine therapeutic application of G-CSF but no clinical benefits have been observed [20].

There is no established information about how best to manage FN and this makes it difficult to adhere to guidelines. For example, nonconventional schedules of short-acting G-CSF (i.e. <6, or even 4, days of administration) are sometimes used based on clinicians’ experience instead of evidence-based data. There is no particular guideline for G-CSF administration in weekly chemotherapy regimens or as prophylaxis for different forms of neutropenia, as happens with the cyclin-dependent kinase inhibitors [3, 21].

According to SEOM and American Society of Clinical Oncology guidelines, antibiotic prophylaxis is not recommended for mild-to-moderate intensity chemotherapy. It can be used only if neutrophils are expected to remain <100 cells/µL for >7 days and there are no other complications [16, 22]. Unfortunately, recommendations for current antimicrobial practice in FN across Europe and Asia are not widely implemented in clinical practice and overuse of antimicrobials and combination therapies have been reported [23]. There have also been reports of incorrect dosing of antimicrobials due to non-compliance with institutional FN management guidelines [24].

### Timing of the use of long- and short-acting formulations

The statements that lacked consensus include: short-acting G-CSFs should be used when long-acting formulations failed to control neutropenia (item 25) and short-acting G-CSFs could be used to accommodate the scheduled chemotherapy cycle (item 27) (see Table 4 in article).

### Toxicity of long- and short-acting formulations

All four statements on the toxicity of the short- and long-acting formulations failed to achieve consensus (see Table 5 in article). They include: differences exist in the incidence of bone pain between patients receiving long- and short-acting G-CSFs (item 28), switching from long- to short-acting formulations is appropriate in case of toxicity the event of toxicity from long-acting formulations (e.g. bone pain; item 29), risk of secondary myeloid neoplasms is increased with short- and long-acting G-CSFs and long-acting G-CSFs could be used if neutrophil leucocytosis develops after the previous administration of G-CSF.

Bone pain is a commonly reported adverse event (AE) associated with G-CSF use [25, 26]. Data from a retrospective study indicate that using a lower dose of pegfilgrastim (3 mg) reduced the incidence of bone pain compared with 6 mg but did not affect absolute granulocyte counts or increase the risk of serious infection [27]. However, no studies to date have compared the incidence of bone pain between patients treated with long- versus short-acting G-CSFs.

Disagreement between the panellists on the increased risk of secondary malignancies with primary G-CSF support could be due to the lack of confirmatory studies. Moreover, secondary malignancies could also result from the leukemogenic effects of high doses of chemotherapeutic agents [28].

### Sphere of application of short- or long-acting agents

Three out of seven statements could not gain consensus (see Table 6 in article). There was no consensus on supportive therapy with G-CSF for patients with a <10% risk of chemotherapy-induced FN (item 35) nor on treatment with long-acting G-CSF after failure to manage FN with short-acting G-CSF in the previous chemotherapy cycle (item 37). There was also no consensus on performing a complete blood count when the blood cells are expected to be at their lowest after a chemotherapy treatment (item 38).

# References

1. Wright JD, Neugut AI, Ananth CV, et al (2013) Deviations from guideline-based therapy for febrile neutropenia in cancer patients and their effect on outcomes. JAMA Intern Med 173(7):559-568. <https://doi.org/10.1001/jamainternmed.2013.2921>

2. Barni S, Lorusso V, Giordano M, et al (2014) A prospective observational study to evaluate G-CSF usage in patients with solid tumors receiving myelosuppressive chemotherapy in Italian clinical oncology practice. Med Oncol 31(1):797. <https://doi.org/10.1007/s12032-013-0797-z>

3. Link H, Kerkmann M, Holtmann L, Ortner P (2019) G-CSF guideline adherence in Germany, an update with a retrospective and representative sample survey. Support Care Cancer 27(4):1459-1469. <https://doi.org/10.1007/s00520-018-4481-x>

4. Fishman ML, Kumar A, Davis S, Shimp W, Hrushesky WJM (2012) Guideline-based peer-to-peer consultation optimizes pegfilgrastim use with no adverse clinical consequences. J Oncol Pract 8(3 Suppl):e14s-17s. <https://doi.org/10.1200/JOP.2012.000540>

5. Danova M, Pronzato P, Ingrasciotta Y, et al (2020) Recent advances in the management of chemotherapy-induced neutropenia: biosimilar granulocyte colony-stimulating factor use in Italy. Future Oncol 16(14):891-897. <https://doi.org/10.2217/fon-2020-0167>

6. Griffiths RI, Barron RL, Gleeson ML, et al (2012) Granulocyte-colony stimulating factor use and medical costs after initial adjuvant chemotherapy in older patients with early-stage breast cancer. Pharmacoeconomics 30(2):103-118. <https://doi.org/10.2165/11589440-000000000-00000>

7. Hübel K, Kron F, Lux MP (2020) Biosimilars in oncology: effects on economy and therapeutic innovations. Eur J Cancer 139:10-19. <https://doi.org/10.1016/j.ejca.2020.07.037>

8. MacDonald K, McBride A, Alrawashdh N, Abraham I (2020) Cost-efficiency and expanded access of prophylaxis for chemotherapy-induced (febrile) neutropenia: economic simulation analysis for the US of conversion from reference pegfilgrastim to biosimilar pegfilgrastim-cbqv. J Med Econ 23(12):1466-1476. <https://doi.org/10.1080/13696998.2020.1833339>

9. Rapoport BL, Aapro M, Paesmans M, et al (2018) Febrile neutropenia (FN) occurrence outside of clinical trials: occurrence and predictive factors in adult patients treated with chemotherapy and an expected moderate FN risk. Rationale and design of a real-world prospective, observational, multinational study. BMC Cancer 18(1):917. <https://doi.org/10.1186/s12885-018-4838-z>

10. Klastersky J, Awada A (2011) Prevention of febrile neutropenia in chemotherapy-treated cancer patients: pegylated versus standard myeloid colony stimulating factors. Do we have a choice? Crit Rev Oncol Hematol 78(1):17-23. <https://doi.org/10.1016/j.critrevonc.2010.02.005>

11. Lyman GH, Kuderer NM (2004) The economics of the colony-stimulating factors in the prevention and treatment of febrile neutropenia. Crit Rev Oncol Hematol 50(2):129-146. <https://doi.org/10.1016/j.critrevonc.2004.01.001>

12. Cornes P, Gascon P, Vulto AG, Aapro M (2020) Biosimilar pegfilgrastim: Improving access and optimising practice to supportive care that enables cure. BioDrugs 34(3):255-263. <https://doi.org/10.1007/s40259-020-00411-4>

13. Mahtani R, Crawford J, Flannery SM, Lawrence T, Schenfeld J, Gawade PL (2021) Prophylactic pegfilgrastim to prevent febrile neutropenia among patients receiving biweekly (Q2W) chemotherapy regimens: a systematic review of efficacy, effectiveness and safety. BMC Cancer 21(1):621. <https://doi.org/10.1186/s12885-021-08258-w>

14. Santini D, Frezza AM, Venditti O, et al (2013) Comment and reply on: pegfilgrastim is safe and effective in the prevention of neutropenia and treatment delays in biweekly regimens. Expert Opin Ther Targets 17(4):473-474. <https://doi.org/10.1517/14728222.2013.773269>

15. Draper AS, Lafollette J, Kim C, Wu CS (2021) Retrospective study evaluating the safety of administering pegfilgrastim on the final day of 5-fluorouracil continuous intravenous infusion. J Oncol Pharm Pract 27(5):1159-1164. <https://doi.org/10.1177/1078155220945771>

16. Carmona-Bayonas A, Jimenez-Fonseca P, de Castro EM, et al (2019) SEOM clinical practice guideline: management and prevention of febrile neutropenia in adults with solid tumors (2018). Clin Transl Oncol 21(1):75-86. <https://doi.org/10.1007/s12094-018-1983-4>

17. Taplitz RA, Kennedy EB, Bow EJ, et al (2018) Outpatient Management of Fever and Neutropenia in Adults Treated for Malignancy: American Society of Clinical Oncology and Infectious Diseases Society of America Clinical Practice Guideline Update. Journal of Clinical Oncology 36(14):1443-1453. <https://doi.org/10.1200/JCO.2017.77.6211>

18. Daniels LM, Durani U, Barreto JN, et al (2019) Impact of time to antibiotic on hospital stay, intensive care unit admission, and mortality in febrile neutropenia. Support Care Cancer 27(11):4171-4177. <https://doi.org/10.1007/s00520-019-04701-8>

19. Mhaskar R, Clark OA, Lyman G, Engel Ayer Botrel T, Morganti Paladini L, Djulbegovic B (2014) Colony-stimulating factors for chemotherapy-induced febrile neutropenia. Cochrane Database Syst Rev 2014(10):Cd003039. <https://doi.org/10.1002/14651858.CD003039.pub2>

20. Hartmann LC, Tschetter LK, Habermann TM, et al (1997) Granulocyte colony-stimulating factor in severe chemotherapy-induced afebrile neutropenia. N Engl J Med 336(25):1776-1780. <https://doi.org/10.1056/nejm199706193362502>

21. Tralongo AC, Antonuzzo A, Pronzato P, et al (2020) Management of chemotherapy-induced neutropenia in patients with cancer: 2019 guidelines of the Italian Medical Oncology Association (AIOM). Tumori 106(4):273-280. <https://doi.org/10.1177/0300891620927093>

22. Flowers CR, Seidenfeld J, Bow EJ, et al (2013) Antimicrobial prophylaxis and outpatient management of fever and neutropenia in adults treated for malignancy: American Society of Clinical Oncology clinical practice guideline. J Clin Oncol 31(6):794-810. <https://doi.org/10.1200/jco.2012.45.8661>

23. Verlinden A, Mikulska M, Knelange NS, et al (2020) Current antimicrobial practice in febrile neutropenia across Europe and Asia: the EBMT Infectious Disease Working Party survey. Bone Marrow Transplant 55(8):1588-1594. <https://doi.org/10.1038/s41409-020-0811-y>

24. Naeem D, Alshamrani MA, Aseeri MA, Khan MA (2018) Prescribing empiric antibiotics for febrile neutropenia: compliance with institutional febrile neutropenia guidelines. Pharmacy (Basel) 6(3). <https://doi.org/10.3390/pharmacy6030083>

25. Dale DC, Crawford J, Klippel Z, et al (2018) A systematic literature review of the efficacy, effectiveness, and safety of filgrastim. Support Care Cancer 26(1):7-20. <https://doi.org/10.1007/s00520-017-3854-x>

26. Lambertini M, Del Mastro L, Bellodi A, Pronzato P (2014) The five "Ws" for bone pain due to the administration of granulocyte-colony stimulating factors (G-CSFs). Crit Rev Oncol Hematol 89(1):112-128. <https://doi.org/10.1016/j.critrevonc.2013.08.006>

27. Lower EE, Charif M, Bartelt M (2018) Reduced dose pegfilgrastim is associated with less bone pain without increased neutropenia: a retrospective study. Cancer Chemother Pharmacol 82(1):165-170. <https://doi.org/10.1007/s00280-018-3607-7>

28. Lyman GH, Yau L, Nakov R, Krendyukov A (2018) Overall survival and risk of second malignancies with cancer chemotherapy and G-CSF support. Ann Oncol 29(9):1903-1910. <https://doi.org/10.1093/annonc/mdy311>
